# Probiotic Properties of *Lactobacillus paracasei* subsp. *paracasei* L1 and Its Growth Performance-Promotion in Chicken by Improving the Intestinal Microflora

**DOI:** 10.3389/fphys.2019.00937

**Published:** 2019-07-25

**Authors:** Yunhe Xu, Yuan Tian, Yunfang Cao, Jianguo Li, Haonan Guo, Yuhong Su, Yumin Tian, Cheng Wang, Tianqi Wang, Lili Zhang

**Affiliations:** ^1^Department of Food Science and Engineering, Jinzhou Medical University, Jinzhou, China; ^2^Tianwang Animal Health Supervision Institute, Jinzhou Economic and Technological Development Zone, Jinzhou, China

**Keywords:** *Lactobacillus*, probiotic, gut microbiota, chicken, growth performance

## Abstract

*Lactobacillus paracasei* subsp. *paracasei* L1 was previously isolated from sweet potato sour liquid. This bacterial species specifically binds onto starch granular surfaces, triggering the enzymatic hydrolysis of raw starch. We investigated the functional and safety properties of strain L1 *in vitro* to establish its probiotic potential, and analyzed its effect on growth performance and intestinal microflora of chicken in feeding experiments. The optimal growth conditions of strain L1 included low pH and high concentrations of bile salts and NaCl. Its 1-, 2-, and 24-h autoaggregation values were 15.8 ± 1.2%, 20.4 ± 2.3%, and 47.2 ± 0.8%, respectively, with the surface hydrophobicity value at 560 nm of 38.1 ± 2.7%. Further, its adhesion rate to Caco-2 cells was 22.37 ± 1.44%. Strain L1 was resistant to erythromycin and azithromycin, but sensitive to other antibiotics tested. For the feeding experiments, 240 chickens with similar weights were randomly divided into a control (C) group and strain L1 (L) group and fed for 8 weeks. Strain L1 promoted the weight gain of chickens in L group. A significant increase in the population size of the two phyla and 23 genera in the small intestine was observed in the presence of strain L1 (*P* < 0.05), with 0 phyla and 4 genera showing significant increase in the cecum (*P* < 0.05). In the small intestine, the abundance of six functional genes at Kyoto Encyclopedia of Genes and Genomes (KEGG) level 2 and 49 genes at KEGG level 3 was significantly increased in group L (*P* < 0.05), with lesser changes noted in the cecum. An increase in the metabolic pathway functions, including enzyme families and the digestive system, was observed in the intestinal microbiota in the L group compared to the C group. However, the other metabolic pathway functions, including metabolism of fatty acid biosynthesis, as well as metabolism of glycerolipids and propanoate, increased in the cecal microbiota of the L group relative to the C group. These changes are most likely related to the changes in the gut microbiota composition. Collectively, strain L1 supplementation may promote growth performance and improve the intestinal microflora in chicken although further studies are needed to confirm this.

## Introduction

In 2006, the European Union prohibited the utilization of AGPs in animal production (European Commission [EC], 2001). This directive has resulted in major problems in animal production such as a significant decrease in growth performance and an increase in the prevalence of diseases that were previously prevented by the use of antibiotics (Babak and Nahashon, 2014; Wielinga et al., 2014; Zou et al., 2016; Al-Khalaifah, 2018). Hence, extensive efforts have been made to develop AGP substitutes as feed additives, which include essential oils, fermented liquid feed, organic acids, probiotics, and prebiotics. Previous studies have shown that probiotics promote growth, thereby enhancing animal production by increasing the intake and conversion rate of feeds and total body weight (Taras et al., 2007; Chaucheyras-Durand and Durand, 2010; Dittoe et al., 2018). Furthermore, probiotics have been shown to aid digestion in animals by improving the absorption of specific essential nutrients (Yu et al., 2008).

Probiotics research has lately focused on LAB, in particular, the bacterial species *Lactobacillus*, *Lactococcus*, and *Bifidobacterium* based on their potential health benefits (Shekh et al., 2016). The term “probiotic” refers to “live microbial species that are beneficial to the host when consumed at sufficient amounts” (FAO/WHO, 2002; O’Connell Motherway et al., 2008; Kotzamanidis et al., 2010). However, despite convincing evidence that certain lactobacilli strains are safe for human utilization as well as confer specific health benefits to the host, these positive effects cannot be applied to other strains in the absence of results from experimentation (FAO/WHO, 2002; Kotzamanidis et al., 2010). Before assessing the *in vivo* probiotic properties of a strain, it is essential to confirm its features relating to safety, survival in the gastrointestinal tract, colonization ability, and other probiotic characteristics.

What is the mechanism by which probiotic bacteria confer host health benefits? Macromolecules of the cell surface of bacteria are major components that interact between probiotic bacteria and its host and involve pattern recognition receptors (PRRs) in gastrointestinal mucosa of the host (Lebeer et al., 2010). In recent years, many probiotic LAB have been found to possess a variety of proteins anchored on the surface or the cell wall, and most of these were enzyme proteins related to carbohydrate metabolism and transport. These proteins play a major role in LAB adhesion onto the intestinal tract and are also responsible for sugar catabolism or degradation of various complex sugars such as lactose or starch (Zhang et al., 2017). The activity of LAB to metabolize carbohydrates is crucial for their colonization of and proliferation in the intestine (Ganzle and Follador, 2012; Duranti et al., 2014; Liu et al., 2015).

*Lactobacillus paracasei* subsp. *paracasei L1* has been isolated from a naturally fermented sour liquid of sweet potato (Zhang et al., 2017). It is surface-anchored via glycoside hydrolase, cell wall peptidoglycan hydrolase, phosphoglycerol kinase, glyceraldehyde-3-phosphate dehydrogenase, enolase, etc., which are related to sugar metabolism and mediate the specific binding of L1 cells to a starch granule. The strain has the ability to hydrolyze raw starch to generate simple carbohydrates, including glucose and lactic acid, that are capable of altering the structural, physical, and chemical features of starch granules (Zhang et al., 2017). The ability of LAB to bind and utilize carbohydrates is important for the colonization of the intestine and promotion of carbohydrate metabolism. By relying on the characteristics of LAB starch metabolism, additional carbon sources are generated for the competitive growth of intestinal microflora, which benefits the competitive growth of LAB in the intestinal tract (Wang et al., 1999; O’Connell Motherway et al., 2008).

Chicken feed contains copious amounts of raw starch. Enhancing the utilization of starch by LAB is greatly important for improving the production performance of chicken and reducing the cost of raising chicken. Moreover, these enzyme proteins on the surface of *L. paracasei* subsp. *paracasei* L1 were also present on the surfaces of many probiotic lactic acid bacteria; therefore, we speculated that *L. paracasei* subsp. *paracasei* L1 has potential probiotic properties. Thus, the aim of the present study was to examine the functional and safety features of strain L1 *in vitro* to determine its potential use as a probiotic. The effect of strain L1 on the growth performance and intestinal microflora of chicken was then evaluated in feeding experiments. The study lays a theoretical foundation for the application of strain L1 in chicken production.

## Materials and Methods

### Materials

Strain L1 has been previously isolated from sour liquid (Zhang et al., 2017). This strain has been deposited to the China General Microbiological Culture Collection Center (CGMCC, No. 4163). Glycerol stocks (30% glycerol, v/v) of the pure culture were stored at −80°C until use. Bacterial cultures were aerobically prepared on a sweet potato juice medium (as described later) at 4°C and then transferred to a fresh medium each month. Prior to analysis, the strain was statically cultured at 30°C for 24 h (until the stationary growth phase) under aerobic conditions. We employed enterocyte-like Caco-2 ECACC 86010202 cells (from colon adenocarcinoma) in simple adhesion assays. PBS, (pH 7.2) was obtained from chemical reagent company (Sigma-Aldrich, St. Louis, MO, United States). All other chemical reagents used in this study were of analytical grade.

The sweet potato juice medium was prepared according to Zhang et al. (2017). Briefly, a sweet potato infusion using 200 g of sliced (washed but unpeeled) sweet potatoes in 1 L of distilled water was boiled for 30 min, and the broth was decanted or strained through a cheesecloth. Then, distilled water was added to the infusion to a total volume of 1 L, to which 20 g of glucose, 2 g of lactose, 5 g of yeast extract, and 5 g of sodium acetate were added. The culture medium was then autoclave-sterilized at 115°C for 15 min (Zhang et al., 2017).

### Acid and Bile Salt Tolerance

Viability of strain L1 was examined according to de Albuquerque et al. (2018). Tolerance to various pH values and concentrations of bile salts was evaluated by inoculating 1-mL aliquots of the strain L1 suspension (grown in sweet potato juice medium for 24 h at 30°C) in 10 mL of sweet potato juice medium at various pH levels (2.0, 2.5, 3.0, or 3.5 by using 1 M HCl) or supplemented with different bile salt concentrations [0.03, 0.3, or 0.5% (w/v)] (Sigma-Aldrich, St. Louis, MO, United States). The suspensions were then incubated at 30°C. We collected 1-mL aliquots of the suspension at different incubation time intervals (1–4 h), and each aliquot was serially diluted in sterile peptone water (0.15 g⋅100 mL^–1^), and streaked onto the MRS agar. The viable cells were manually counted and expressed in terms of log cfu⋅mL^–1^. For the control, strain L1 cells were cultivated in sweet potato juice medium (pH 7, adjusted with 1 M NaOH) in the absence of bile salts (de Albuquerque et al., 2018).

### NaCl Tolerance

The strain L1 cultures (grown in sweet potato juice medium for 24 h at 30°C) were transferred (5%, v/v) into fresh sweet potato juice medium containing 1, 2, 3, 4, or 5% (w/v) NaCl or a fresh sweet potato juice medium without NaCl (control) and incubated at 30°C. Viable cells in the medium with and without NaCl were counted after incubating for 24 h and expressed in terms of log cfu⋅mL^–1^.

### Autoaggregation Assay

To assess autoaggregation capacity, the strain L1 cells grown in sweet potato juice medium (for 24 h at 30°C) were harvested by centrifugation at 3,000 × *g* for 10 min at 20°C, washed with PBS twice, and then resuspended in PBS to an OD_660_ of 0.3. After incubating at 37°C for 60 min, the OD_660_ value was again measured. Autoaggregation was calculated using the following equation (1):

(1) $$\mathrm{Autoaggregation}\left(\%\right)=\frac{{\mathrm{OD}}_{0}-{\mathrm{OD}}_{60}}{{\mathrm{OD}}_{0}}\times 100\%$$

where OD_0_ is the initial OD value, and OD_60_ is the OD value after incubating for 60 min (de Albuquerque et al., 2018).

### Cell Surface Hydrophobicity

Bacterial adhesion onto hydrocarbon-like toluene was assessed as described by Iñiguez-Palomares et al. (2007). The strain L1 cells (5-mL suspension) were harvested in triplicate via centrifugation at 3,000 × *g* for 15 min, washed with PBS (pH 7.2) twice, and then re-suspended in the same buffer to a density of approximately 10^8^ cfu⋅mL^–1^ (OD_560_; A). Then, 4 mL of each suspension were mixed with 1.2 mL of toluene. After incubating for 10 min, the bacterial suspension was then thoroughly mixed with toluene by vortexing for 2 min. Then, the OD (A_0_) of the aqueous phase was determined at a wavelength of 560 nm. The hydrophobicity percentage (H) was estimated as the equation (2):

(2) $$H\left(\%\right)=\frac{A-\mathrm{A0}}{A}\times 100\%$$

where A and A_0_ are the absorbance values that were measured before and after toluene extraction, respectively (Iñiguez-Palomares et al., 2007).

### Bacterial Adhesion Onto Caco-2 Cells

Human colon cancer Caco-2 cells were routinely cultured in Dulbecco’s modified Eagle’s medium (DMEM) containing 10% (w/v) fetal bovine serum and 1% (w/v) antibiotic solution (100 μg⋅mL^–1^ penicillin and 100 μg⋅mL^–1^ streptomycin). The cells were cultured in flasks at 37°C in 5% CO_2_ atmosphere. The Caco-2 cells were inoculated into a six-well cell culture plate at a density of 10^5^ cells per well for cell fusion, then cultured for 20 days and employed in the adhesion assay. The cell culture medium was replaced with fresh DMEM supplemented with 2% (w/v) fetal bovine serum without the antibiotics for at least 1 h prior to the adhesion assay. A *Lactobacillus* suspension (10^8^ cfu⋅mL^–1^ in PBS) was added to each well of the tissue culture plate and cultured for 3 h at 37°C and 5% CO_2_ atmosphere. The plate was then washed thrice with 1 mL of PBS to remove non-adhering bacteria. The cells were then incubated with Triton X-100 (0.05%) for 10 min. The lysate was diluted, followed by coating with the appropriate diluent on MRS agar. Adhesivity was expressed as the percentage of bacteria that adhered onto the Caco-2 cells and the initial number of bacteria (Pisano et al., 2014).

### Safety Assessment

Strain L1 antibiotic susceptibility was assessed using the disc diffusion method as described by the Clinical and Laboratory Standards Institute (Clinical and Laboratory Standard Institute, 2009). However, the Mueller-Hinton agar was substituted with MRS agar in the assay. We tested the following antibiotics (Oxoid): amoxicillin/clavulanic acid (30 μg), azithromycin (15 μg), cefotaxime (30 μg), ciprofloxacin (5 μg), erythromycin (15 μg), gentamicin (10 μg), kanamycin (30 μg), norfloxacin (5 μg), penicillin G (10 μg), rifampicin (30 μg), streptomycin (10 μg), teicoplanin (30 μg), tetracycline (30 μg), and vancomycin (30 μg). We placed an antibiotic disc on the MRSA agar plate after spreading an overnight strain L1 culture (0.1 mL) using an antibiotic disc dispenser, and the plates incubated for 24 h at 30°C. We measured the diameters of the bacterium-free zones, and the results were expressed as resistance based on the interpretative criteria established by the Clinical & Laboratory Standards Institute. Plasmid extraction was done using GeneJET plasmid miniprep kit (Thermo Fisher Scientific, United States).

The amino acid decarboxylating activity of strain L1 (L-histidine, L-lysine, L-ornithine, and L-tyrosinel Sigma-Aldrich) was determined according to Bover-Cid and Holzapfel (1999). Briefly, the strain L1 culture was inoculated (2%, v/v) in a decarboxylase medium with or without amino acids (the control). After incubating at 30°C for 72 h, we confirmed biogenic amine production based on color changes in relation to amine formation. A positive result was established when a change in the color of the medium was detected (Bover-Cid and Holzapfel, 1999).

Hemolytic activity was assessed by streaking the cells onto Columbia blood agar plates that were supplemented with 5% defibrinated sheep blood, and then incubated for 48 h at 37°C. After incubating, the hemolytic reaction was evaluated based on the presence of a clear zone of hydrolysis surrounding the colonies (β-hemolysis), partial hydrolysis, as well as a greenish zone (α-hemolysis) or no reaction (γ-hemolysis). Positive controls were prepared using *Staphylococcus aureus* ATCC 25923 cells.

### Chickens, Treatment, and Sampling

A total of 1000 1-day-old Dagu × Xianju chickens were used in this study. The chickens were maintained in plastic mesh floors (situated 80 cm aboveground) for 8 weeks. The chickens were given feed and water *ad libitum*. The temperature of the house was controlled at 35°C during the 1st week and then decreased by 2°C per week to a final temperature of 23°C. Approximately eight weeks later, the chickens were weighed, and around 240 chickens with similar weights were randomly assigned to the control (C) and strain L1 (L) groups. Each group comprised three replicates with 40 birds (50% males and 50% females) per replicate. The chickens were raised in their cages (50 cm × 50 cm × 50 cm, situated 80 cm aboveground). The temperature of the chicken house was set at 23°C. The chickens were given feed and water *ad libitum* (Xu Y. et al., 2016). The initial difference in body weight was not significant between the two groups (*P* > 0.05). The two groups were provided with the same basal diet (Supplementary Table S1) and subjected to similar environmental factors. The two groups were fed with mash diets, namely, the C group was given a basal diet and liquid medium, whereas the L group received a basal diet and 1 × 10^6^ cfu of strain L1⋅g^–1^. Chickens were weighed, and feed intake was recorded on the morning of days 91 and 112. The average daily gain (ADG), average daily feed intake (ADFI), and feed conversion ratio (FCR) were also calculated. When the chickens reached 16 weeks of age, these were each weighed. Three chickens that were deemed representative of the average weight were randomly picked out from each group and killed. The contents of the small intestine (the posterior duodenum and anterior jejunum) and cecum were collected, flash-frozen in liquid nitrogen, and used in DNA extraction and PCR analysis. The samples were classified into four groups as follows: the XC group (small intestines from group C), XL group (small intestine sample from group L), DC group (the cecum sample from group C), and DL group (the cecum sample from group L).

### Feed Preparation

The number of viable strain L1 cells was assessed using plate counting after culturing the cells in sweet potato juice medium at 30°C for 24 h. We performed feed supplementation prior to each feeding as follows: approximately 10 mL of the strain L1 liquid culture (only liquid medium for C group) were thoroughly mixed with 1000 g of the corresponding diet to attain a strain L1 density of 1 × 10^6^ cfu⋅g^–1^ after mixing (Wang et al., 2017).

### *16S rRNA* Sequencing of Gut Microbes

Microbial genomic DNA was isolated from the cecal content samples with a TIANGEN DNA stool mini kit (TIANGEN, cat#DP328), following the manufacturer’s recommendations. The variable region of *16S rRNA* V3–V4 was PCR amplified with universal primers 338F (5′-ACTCCTACGGGAGGCAGCAG-3′) and 806R (5′-GGACTACHVGGGTWTCTAAT-3′) (Xu N. et al., 2016). The PCR products were purified with a QIAGEN quick gel extraction kit (QIAGEN, Cat # 28706). The PCR products from each sample were employed in the construction of a sequencing library with an Illumina TruSeq DNA sample preparation kit (the library was constructed with a TruSeq Library construction kit). For each sample, barcoded V3–V4 PCR amplicons were sequenced on an Illumina MiSeq PE300 platform.

Sequence reads were discarded when the sequence length was <150 bp, if the average Phred score was <20, if these contained ambiguous bases, if the homopolymer run >6, or if there were primer mismatches. The sequences that passed quality filtering were assembled using Flash^1^, which required an overlap of reads 1 and read of ≥10 bp, without any mismatches. We discarded any reads that could not be assembled. Chimera sequences were also discarded using UCHIME in MOTHUR (version 1.31.2^2^). Amplification and sequencing of the *16S rRNA* V3–V4 variable region was completed by Shanghai Majorbio Bio-pharm Technology Co., Ltd. (Shanghai, China).

### Operational Taxonomic Unit (OTU) Clustering

Sequence clustering was conducted with the uclust algorithm in QIIME^3^, and clustered into OTUs. The longest sequence in every cluster was chosen as the representative. The taxonomy of every OTU was assigned using BLAST-searching for the representative sequence in the Greengenes reference database (release 13.8^4^). Unknown archaeal or eukaryotic sequences were filtered out. The Ace, Chao, Shannon, and Simpson indices were computed using the summary.single command in MOTHUR. A Venn diagram of between-group OTUs was constructed in R. We compared the relative abundance of OTUs or taxa among samples.

### Microbial Function Prediction

Functional genes were predicted with PICRUSt based on the abundance at the OTU level. The OTUs were mapped to the gg13.5 database at a 97% similarity with the QIIME command “pick_closed_otus.” OTU abundance was automatically normalized with the *16S rRNA* gene copy numbers from known bacterial genomes of the Integrated Microbial Genomes database. The predicted genes and their function were annotated with the KEGG database, then differences among groups were compared to the free online platform Majorbio I-Sanger Cloud Platform^5^. Two-side Welch’s *t*-test and Benjamini–Hochberg FDR correction were used in two-group analysis. The relative abundance of the KEGG metabolic pathways was designated as the metabolic profile.

### Statistical Analysis

We statistically analyzed the diversity index data using ANOVA, and significant differences between group means were assessed using the Duncan test. Growth performance and abundance at the phylum and genus levels between groups were statistically evaluated with the *t*-test. Diversity indices and growth performance were expressed as the mean ± standard error (SE). We generated PCoA plots for sequence read abundance with Vegan as implemented in R. All statistical analyses were conducted using SPSS 16.0.

## Results

### *In vitro* Characterization of Strain L1

Strain L1 was tolerant to acidic and biliary conditions. The viable counts slightly decrease upon exposure to low pH or high bile salt concentrations. After incubating for 3 h in a medium at pH 2, strain L1 had a survival rate of 98.73%, whereas after 4 h incubating in a medium supplemented with bile salts (0.5 g⋅mL^–1^), it was 98.35%. These findings indicated that strain L1 can normally grow in these conditions with high viability (viability: 7.5–8 log cfu⋅mL^–1^) (Tables 1, 2). Hence, the strain could meet the concentration requirement of probiotics for use in animals.

**TABLE 1:** Counts of strain L1 exposed to different pH values for different time periods (*n* = 3).

**pH**	**Growth of strain L1 (log cfu mL^–1^)**			
	**0 h**	**1 h**	**2 h**	**3 h**
7.0 (control)	7.90 ± 0.66^a^	8.52 ± 0.43^b^	8.77 ± 0.28^b^	8.93 ± 0.37^b^
3.5	7.88 ± 0.43^a^	7.88 ± 0.64^a^	7.91 ± 0.54^a^	7.94 ± 0.11^a^
3.0	7.88 ± 0.28^a^	7.88 ± 0.45^a^	7.88 ± 0.32^a^	7.90 ± 0.87^a^
2.5	7.89 ± 0.27^a^	7.86 ± 0.31^a^	7.86 ± 0.42^a^	7.86 ± 0.57^a^
2.0	7.88 ± 0.51^a^	7.79 ± 0.23^a^	7.79 ± 0.02^a^	7.78 ± 0.16^a^

*The data are presented as the mean ± standard error. Means within a column followed by a different lowercase letter are significantly different (*P* < 0.05).*

**TABLE 2:** Counts of strain L1 exposed to different bile salt concentrations (w/v) for different time periods (*n* = 3).

**Bile salt concentration (g mL^–1^)**	**Growth of strain L1 (log cfu mL^–1^)**				
	**0 h**	**1 h**	**2 h**	**3 h**	**4 h**
0 (control)	7.89 ± 0.25^a^	8.59 ± 0.10^b^	8.70 ± 0.45^b^	8.88 ± 0.08^b^	8.99 ± 0.56^b^
0.03	7.88 ± 0.54^a^	7.85 ± 0.55^a^	7.86 ± 0.73^a^	7.86 ± 0.33^a^	7.87 ± 0.44^a^
0.3	7.88 ± 0.08^a^	7.83 ± 0.13^a^	7.81 ± 0.29^a^	7.82 ± 0.72^a^	7.82 ± 0.33^a^
0.5	7.88 ± 0.44^a^	7.79 ± 0.53^a^	7.76 ± 0.34^a^	7.76 ± 0.13^a^	7.75 ± 0.48^a^

*The data are presented as the mean ± standard error. Means within a column followed by a different lowercase letter are significantly different (*P* < 0.05).*

**FIGURE 1:**
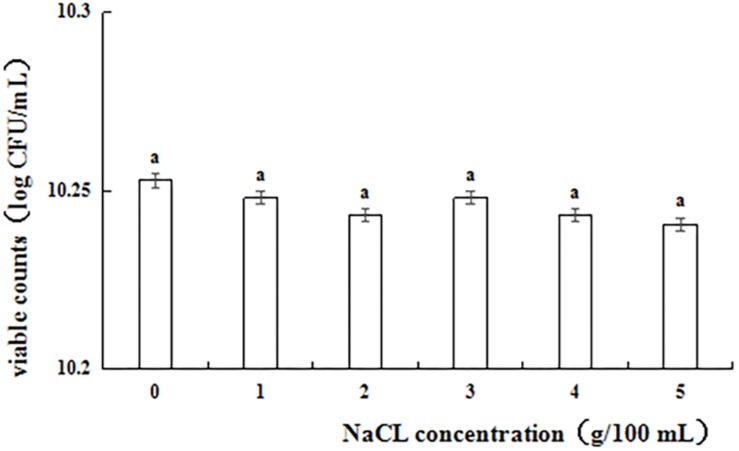
Viable counts of strain L1 in the presence of different salt concentrations. Each column represents the mean of three replicates. The bars represent the standard deviation. Columns with same letter indicate no statistical significance (*p* > 0.05).

Figure 1 shows the results of the NaCl tolerance test. Strain L1 exhibited good tolerance to 1–5% NaCl, with viability within the range of 10.24–10.26 log cfu⋅mL^–1^. After incubating for 24 h, the viable counts of strain L1 in an NaCl-containing medium decreased slightly compared with those in an NaCl-free medium. However, even at NaCl concentration of 5 g 100 mL^–1^, the cell survival rate was as high as 97.2%, with the viable counts in the NaCl-containing medium remaining above 10 log cfu mL^–1^. Thus, strain L1 is highly tolerant to salt, enabling it to withstand the adverse effects of high osmotic pressure in the high-salt environment of the gastrointestinal tract and maintain the relative balance of osmotic pressure under such conditions.

Strain L1 showed good autoaggregation and hydrophobicity properties. After 1, 2, and 24 h, the autoaggregation values were 15.8 ± 1.2%, 20.4 ± 2.3%, and 47.2 ± 0.8%, respectively. Further, at 560 nm, the surface hydrophobicity value was 38.1 ± 2.7%.

**FIGURE 2:**
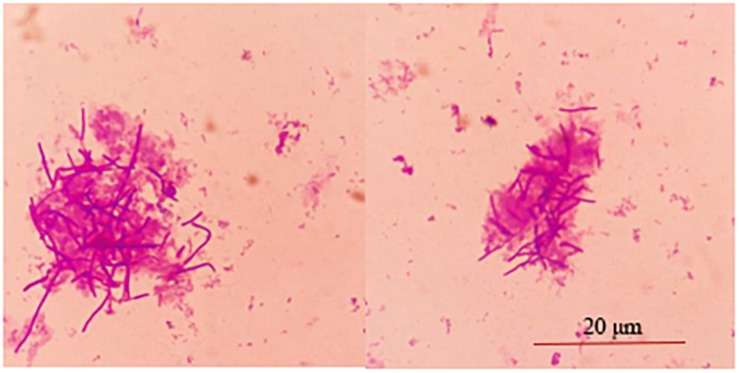
Adhesion of strain L1 to Caco-2 cells. The cells were gram-stained and observed under a microscope imaging system (Olympus DP73) (×1000).

The adhesion of strain L1 onto Caco-2 cells was evaluated microscopically and by plate colony counting. Microscopic observation focused on observing the adhesion of cells, while the plate count was focused on quantifying the adherent cells. Figure 2 shows that approximately 30 ± 2.8 strain L1 cells had adhered onto the surface of Caco-2 cells. In addition, plate colony counts indicated showed that the adhesion rate of strain L1 onto the Caco-2 cells was 22.37 ± 1.44%.

Fourteen antibiotics from different families were investigated (Table 3). Strain L1 showed resistance to erythromycin and azithromycin, but sensitivity to other antibiotics tested. Importantly, it did not contain natural plasmid DNA (data not shown).

**TABLE 3:** Antibiotic susceptibility of strain L1.

**Strain**	Antibiotic tested^*^													
	AMC	P	CTX	VA	TEC	TE	ST	K	GM	E	AZM	CIP	NOR	RD
**Strain L1**	S	S	S	S	S	S	S	S	S	R	R	S	S	S

^*^Antibiotics: AMC, amoxicillin/clavulanic acid; P, penicillin G; CTX, cefotaxime; VA, vancomycin; TEC, teicoplanin; TE, tetracycline; ST, streptomycin; K, kanamycin; GM, gentamicin; E, erythromycin; AZM, azithromycin; CIP, ciprofloxacin; NOR, norfloxacin; RD, rifampicin. R, resistant; S, sensitive. Each experiment was repeated three times.

**FIGURE 3:**
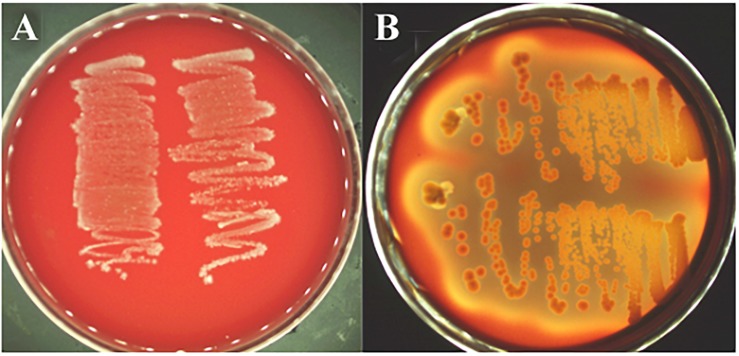
Hemolysis test. **(A)** *L. paracasei* subsp. *paracasei* L1, no reaction (γ-hemolysis); **(B)** *S. aureus* ATCC 25923, a clear zone of hydrolysis surrounding the colonies (β-hemolysis).

The decarboxylase activity of strain L1 was not observed as no culture medium color change was detected, which suggests that no biogenic amines were produced (data not shown). Furthermore, the strain did not show any α- and β-haemolytic activity when cultured on Columbia sheep blood agar, indicating that no hemotoxin was produced (Figure 3).

### OTU Clustering and Annotation

**FIGURE 4:**
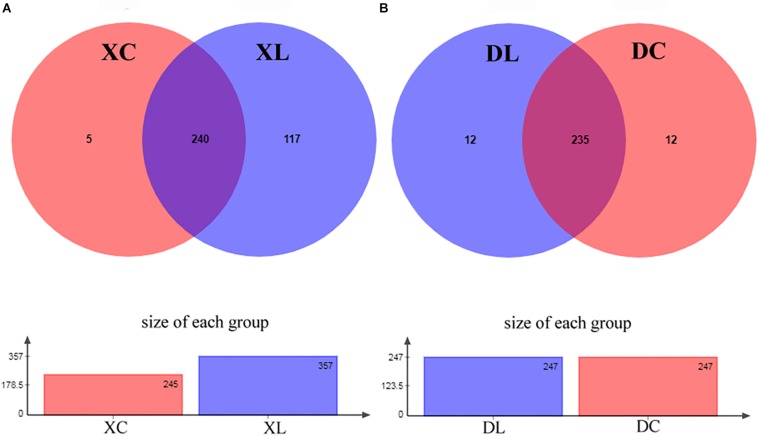
Analysis of OTUs shared by different groups. **(A)** In the experiment, 245 and 357 OTUs in the XC and XL groups, respectively, were identified. The analysis revealed that the XC and XL groups shared 240 OTUs. The overall richness was 362 OTUs. **(B)** In the experiment, 247 OTUs each were identified in the DC and DL groups. The analysis revealed that the DC and DL groups shared 235 OTUs. The overall richness was 259 OTUs.

The ability of strain L1 to act as a probiotic was next investigated through feeding experiments employing chickens fed a diet supplemented with strain L1. After 8 weeks of feeding, the intestinal contents of chickens in each group were collected. Microbial genomic DNA was extracted, followed by the amplification of the variable region of *16S rRNA* V3–V4 and sequencing on an Illumina MiSeq PE300 platform. Trimmed and assembled sequences were then clustered at 97% similarity, as detailed in the Methods, and a total of 378 OTUs were identified by database alignment using BLAST-searching in QIIME. The following OTU numbers were obtained from each group: 245 in the XC group, 357 in the XL group, 247 in the DC group, and 247 in the DL group (Figure 4). Figure 4A shows 117 unique OTUs in XL group and 5 unique OTUs in XC group. The total richness in the X groups (XC and XL groups) was 364 OTUs, but it was 259 OTUs in the D groups (DC and DL groups). The number of OTUs in each group did not change in the D groups; however, this number increased in the X groups after feeding of strain L1. The microbial diversity in the X groups significantly changed. The Chao and Ace indices of the XL group significantly increased (*P* < 0.05) compared to the three other groups. No significant difference (*P* > 0.05) in the Shannon and Simpson of the small intestines was observed between the XC and XL groups; the same trend was observed in the cecum. These findings indicated that the richness of the small intestinal microbes in the XL group was greater compared with the three other groups (Table 4).

**TABLE 4:** Diversity index.

**Group (*n* = 3)**	**Small intestine**		**Cecum**	
	**XC**	**XL**	**DC**	**DL**
**Chao**	361.39 ± 72.97^a^	689.55 ± 58.20^b^	393.46 ± 11.79^a^	377.75 ± 23.91^a^
**ACE**	363.93 ± 76.65^a^	667.77 ± 56.16^b^	395.39 ± 12.93^a^	366.77 ± 27.91^a^
**Shannon**	2.63 ± 0.15^a^	3.02 ± 0.57^a,b^	3.79 ± 0.14^b^	3.85 ± 0.16^b^
**Simpson**	0.150 ± 0.017^a,b^	0.251 ± 0.09^b^	0.06 ± 0.013^a^	0.049 ± 0.006^a^

*The data are presented as the mean ± standard error. Means with the same superscript letter within the same row are not significantly different. Different lowercase letters indicate significance at *P* < 0.05.*

### Differences in the Growth Performance and Intestinal Microbiota in Chicken Associated With the Feeding of Strain L1

In the current study, the growth performance of chickens in different groups was obviously different. At the first stage of the experiment (9–13 weeks), the ADFI of chickens in group L was relatively higher (*P* < 0.05) compared with group C. At the second stage of the experiment (14–16 weeks), group L show better ADG and FCR than that of group C (*P* < 0.05) (Table 5).

**TABLE 5:** Effects of *L. paracasei* subsp. *paracasei* L1 on chicken growth performance.

**Parameter^1^**	**Group**	
	**C**	**L**
**Weeks 9–13 (*n* = 120)**		
ADFI, g/day	102.17 ± 0.68^a^	107.63 ± 0.99^b^
ADG, g/day	27.15 ± 0.41^a^	28.39 ± 0.63^a^
FCR	3.76 ± 0.07^a^	3.79 ± 0.07^a^
**Weeks 14–16 (*n* = 120)**		
ADFI, g/day	129.11 ± 0.22^a^	130.36 ± 0.68^a^
ADG, g/day	25.48 ± 0.20^a^	27.10 ± 0.23^b^
FCR	5.06 ± 0.04^b^	4.81 ± 0.07^a^

*The data are presented as the mean ± standard error. Means within the same row denoted by different lowercase letters are significantly different at *P* < 0.05. ^1^ADFI, average daily feed intake; ADG, average daily gain; FCR, feed conversion ratio (ADFI/ADG).*

**FIGURE 5:**
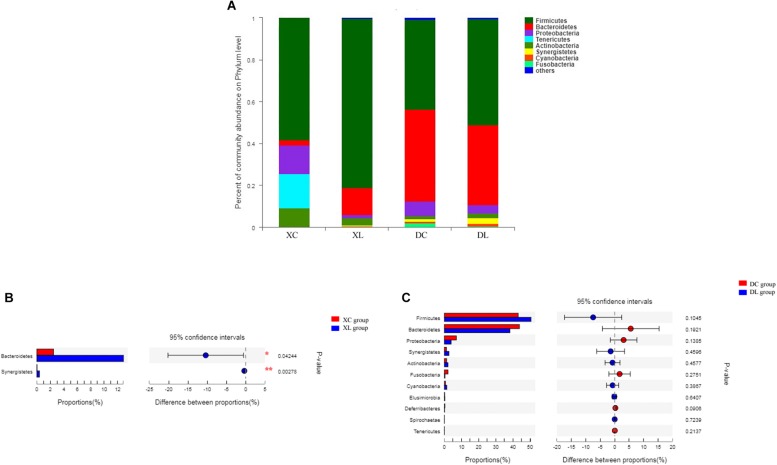
Distribution of the intestinal microbiota composition of chicken at the phylum level. **(A)** The respective proportions of each phylum in the XC, XL, DC, and DL groups. **(B)** Comparison of microbiota abundance in the XC and XL groups. **(C)** Comparison of microbiota abundance in the DC and DL groups.

Twelve phyla were shared by the 12 samples. Firmicutes (>58%) were the dominant bacteria in the small intestine. Firmicutes (>43%) and Bacteroidetes (>38%) were the dominant bacteria in the cecum. Feeding strain L1 greatly impacted the composition of small intestinal microbiota. As shown in Figure 5, the feeding of strain L1 decreased the proportion of Proteobacteria, Actinobacteria, and Tenericutes in the small intestine (*P* > 0.05), increased the proportion of Firmicutes (*P* > 0.05) (Figure 5A), and significantly increased the proportion of Bacteroidetes and Synergistetes (*P* < 0.05) (Figure 5B). Figures 5A,C show that feeding of strain L1 resulted in minimal effects on the cecal microbiota at the phylum level.

**FIGURE 6:**
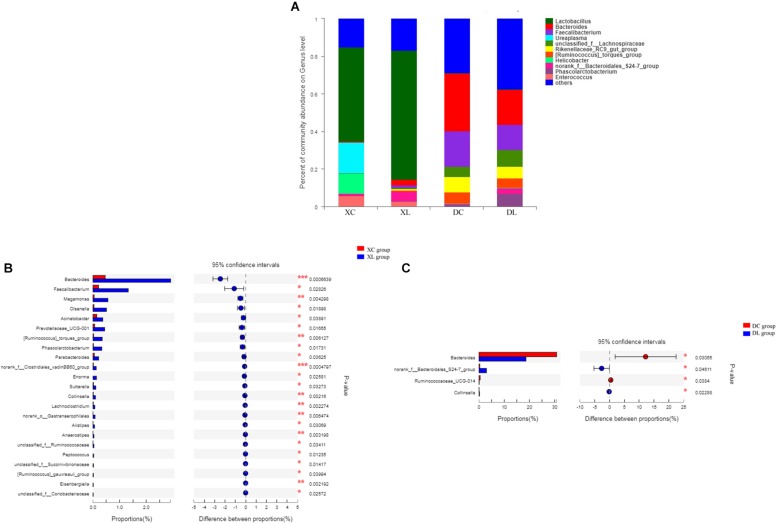
Distribution of the intestinal microbiota composition of chicken at the genus level. **(A)** The respective proportions of each genus in the XC, XL, DC, and DL groups are shown. **(B)** Comparison of microbiota abundance in the XC and XL groups. **(C)** Comparison of microbiota abundance in the DC and DL groups.

At the genus level, we detected 130 genera. *Lactobacillus* (>50%) was the dominant bacterium in small intestine, while *Bacteroides* (> 18%) and *Faecalibacterium* (>13%) were the dominant bacteria in the cecum. Feeding strain L1 resulted in an increase in the proportion of *Lactobacillus* (*P* > 0.05), *Bacteroides* (*P* < 0.05), and *Faecalibacterium* (*P* < 0.05), and decreased the proportion of *Ureaplasma*, *Helicobacter*, and *Enterococcus* (*P* > 0.05) in the small intestine. The cecal abundance of norank_f_Bacteroidales_S24-7_group had increased (*P* < 0.05), and that of *Bacteroides* was significantly (*P* < 0.05) lower with strain L1 feeding (Figure 6).

**FIGURE 7:**
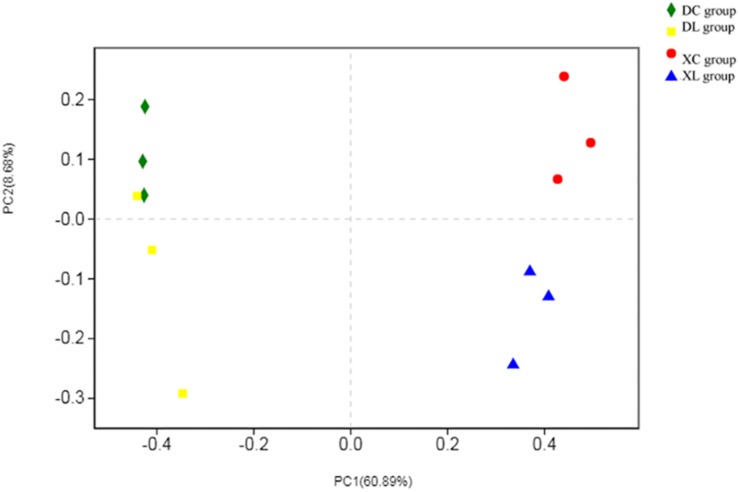
PCoA performed at the OTU level based on unweighted UniFrac distance for all samples. Each group is represented by a different color and shape.

Principal coordinate analysis indicated differences in microbial distribution among the four groups. The distribution markedly differed among groups that had or had not been fed with strain L1 (Figure 7). One group of microorganisms predominated in the L groups (XL and DL groups), whereas another predominated in the C groups (XC and DC group). Correlation analysis showed that the small intestine microbiota in the XC group varied from those in the XL group (0.640). However, the cecal microbiota in the DC group were the same as those in the DL group (0.912) (Table 6). These observations demonstrated that strain L1 greatly impacted the microbiota of the small intestine but had little effect on cecal microbiota.

**TABLE 6:** Correlation of genus abundance between groups.

	**XC (*n* = 3)**	**DC (*n* = 3)**	**DL (*n* = 3)**
XL (*n* = 3)	0.640	0.230	0.312
XC (*n* = 3)		–0.135	–0.150
DC (*n* = 3)			0.912

*Three samples from each group were used to calculate the correlation.*

**FIGURE 8:**
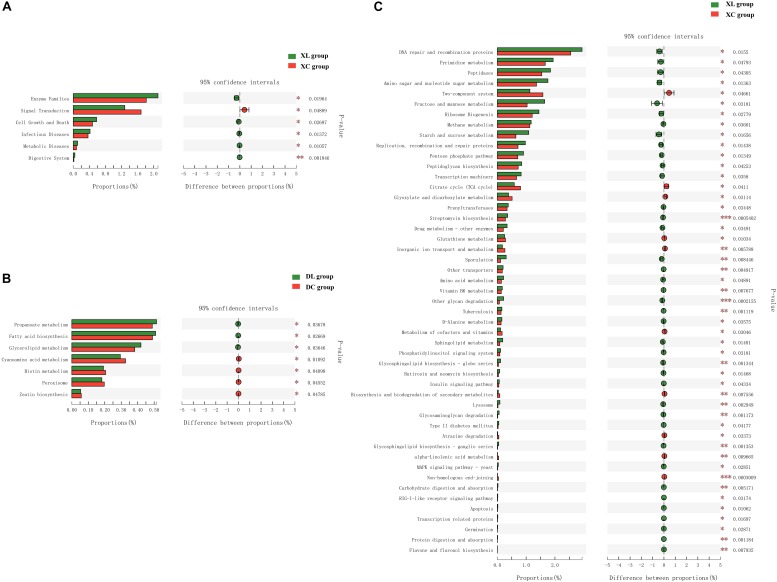
Mean proportion and differences in the predicted functional metagenomes of the intestinal microbiota. Comparison of the functional pathways of microbes in the XC and XL groups at KEGG level 2 **(A)**; the DC and DL groups at KEGG level 3 **(B)**; and the XC and XL groups at KEGG level 3 **(C)**.

Microbial functional analysis using PICTUSt was performed to assess the functions of the microbiota in the C and L groups. At KEGG level 2, in the cecum, no differences in gene abundances between the experimental and control groups were apparent (Supplementary Figure S1). However, in the small intestine, significant differences in the abundances of six functional genes were observed (Figure 8A). The small intestine microbiota in the XL group exhibited a wider range of functions that are involved in metabolic pathways, which include enzyme families (*P* < 0.05) as well as the digestive system (*P* < 0.01), than those in the XC group. At KEGG level 3, in the small intestine, significant differences in the abundance of 49 genes were noted between the XL and XC groups (Figure 8C). The small intestine microbiota in the XL group exhibited greater abundance of functions that were involved in carbohydrate and protein metabolic pathways, including metabolism of starch and sucrose, fructose, and mannose, amino sugars, and nucleotide sugars, degradation of other glycans, insulin signaling pathway, glycosaminoglycan degradation, digestion and absorption of carbohydrates, and digestion and absorption of proteins, than those in the XC group. In the cecum, significant differences in abundance of only seven genes were noted (Figure 8B). Cecal microbiota in the DL group exhibited greater abundance of functions that were involved in metabolic pathways, including fatty acid biosynthesis, glycerolipid metabolism, and propanoate metabolism, than those in the DC group.

## Discussion

The present study assessed the *in vitro* features of a potential probiotic, *L. paracasei* strain L1, and evaluated its weight-stimulating ability using chicken-feeding experiments. Our results showed that strain L1 may indeed be considered a good probiotic candidate.

To be qualified as a probiotic, the microbial candidate must possess specific functional and safety properties, including acid and bile salt tolerance, adhesion capacity, haemolytic activity, and susceptibility to antibiotics (Dowarah et al., 2018). Acid and bile salt tolerance is an essential criteria in identifying probiotic strains, as these influence their survival in the gastrointestinal tract (Saarela et al., 2000). During their passage through the stomach, these probiotic microbes need to survive in as low as pH 3 before reaching the lower digestive tract, and also must remain viable for 4 h or more (Ouwehand et al., 1999). Consequently, strain L1 exhibited good probiotic features as it showed considerable growth at acidic pH (2.0). Du Toit et al. (1998) revealed that *L. reuteri* BFE1058 and *L. johnsonii* BFE1061 isolated from pig fecal material can grow at low pH levels for 6 h at 37°C (Du Toit et al., 1998). Cultures using *L. lactis* and *Enterococcus faecium* have better tolerance to low pH than *Lactobacillus casei* and *Pediococcus acidilactici*, and thus they were fed as probiotics to weaned piglets (Guerra et al., 2007). For an effective probiotic culture, LAB should remain viable in the presence of 0.3% bile salts. *L. plantarum* ZlP001 isolate from the gastrointestinal tract of a weaned piglet exhibited 85.3, 61.4, and 9.4% tolerance to growth medium supplemented with 0.1, 0.3, and 0.5% bile salts, respectively (Wang et al., 2011). Here, strain L1 exhibited 99.8, 99.2, and 98.3% tolerance upon respective exposure to 0.03, 0.3, and 0.5% bile salts for 4 h. The adaptation to bile salts has been shown to be related to alterations in carbohydrate fermentation and glycosidase activity (Taheri et al., 2009); exopolysaccharide production (Petsuriyawong and Khunajakr, 2011; Shazali et al., 2014); the composition of membrane proteins and fatty acids (Fairbrother et al., 2005); and enhanced adhesion to human mucus as well as inhibition of pathogen adhesion (Cho et al., 2009; Venkatesan et al., 2012). Further, strain L1 showed high tolerance to all NaCl concentrations tested (Figure 1), which can enable it to withstand the adverse effects of high osmotic pressure in the high-salt environment of the gastrointestinal tract and maintain a relative balance of osmotic pressure under these conditions.

Hydrophobicity is essential to adhesion to enterocyte-like cells and autoagglutination. Adhesive strains exhibit high levels of hydrophobicity, and the extent of adherence apparently depends on the surface potential (Pérez et al., 1998; Juarez Tomas et al., 2005). Al Kassaa et al. (2014) revealed that CMUL57 (*Lactobacillus gasseri*), CMUL67 (*Lactobacillus acidophilus*), and CMUL140 (*Lactobacillus plantarum*) were most hydrophobic strains among ones screened by the authors; interestingly, these strains also exhibit the greatest autoaggregation ability (Al Kassaa et al., 2014). Adherence of lactobacilli onto epithelial cells and biofilm formation has been associated with cell autoaggregation and surface hydrophobicity (Dunne et al., 2001). In the current study, strain L1 showed good autoaggregation and hydrophobicity abilities. At 560 nm, its surface hydrophobicity value was 38.1 ± 2.7%. Further, the autoaggregation ability increased notably with incubation time. After 1, 2, and 24 h, the autoaggregation values were 15.8 ± 1.2%, 20.4 ± 2.3%, and 47.2 ± 0.8%, respectively. Shekh et al. (2016) previously described the autoaggregation of 10 LAB strains. Autoaggregation after 2 h, 4 h, and 24 h was respectively within the range of 1–19%, 5–46%, and 33–92% (Shekh et al., 2016). Das et al. (2016) reported the hydrophobicity values of *L. casei* SB71, SB73, and SB93 as 22.2 ± 0.8%, 22 ± 1.5%, and 25 ± 2.5%, respectively (Das et al., 2016). Hence, the hydrophobicity of strain L1 was higher than that described in earlier studies (Pelletier et al., 1997; Vinderola and Reinheimer, 2003).

An important feature of probiotics is their ability to adhere to the intestinal epithelial layer that prevents their elimination through peristalsis. Furthermore, adhesion is a prerequisite for colonization (Forestier et al., 2001) and influences the competitive exclusion of enteropathogens (Lee et al., 2003), stimulation of the immune system (Schiffrin et al., 1995), as well as antagonistic activity against enteropathogens (Coconnier et al., 1993). The adhesion ability of *Bifidobacterium* and *Lactobacillus* strains differs with the *in vitro* method employed (Laparra and Sanz, 2009). Here, Caco-2 cells were employed to study the adhesion of strain L1 cells. Plate colony counting showed that the adhesion rate of strain L1 onto Caco-2 cells was 22.37 ± 1.44%. Pisano et al. (2014) revealed that these strains can adhere onto Caco-2 cells to various degrees (ranging from 3 to 20%), thus confirming that adhesion is strain-specific (Pisano et al., 2014). Although the findings of the *in vitro* studies are not directly utilized in *in vivo* situations, these support the association between the two factors (Crociani et al., 1995).

One of the properties that are crucial for identifying LAB as potential probiotics is their safety for human consumption. The antibiotic susceptibility of strain L1 to 14 antibiotics was evaluated by disc diffusion on MRS agar plates (Table 3). Strain L1 exhibited susceptibility to most antibiotics tested, as well as resistance to erythromycin and azithromycin. These results are with the findings involving *L. plantarum* and *L. paracasei* strains (Lavilla-Lerma et al., 2013; Solieri et al., 2014), although other research detected variations in resistance to tetracycline (Georgieva et al., 2008; Comunian et al., 2010). The observed resistance to certain antibiotics indicates that strain L1 will not be affected by therapies involving these antibiotics and thus may facilitate in maintaining the natural balance of intestinal microflora while undergoing antibiotic treatment (Salminen et al., 1998). Natural bacterial resistance to antibiotics is not deemed as a risk to the health of animals or humans (Al Kassaa et al., 2014). Abriouel et al. (2015) analyzed patterns of phenotypic and genotypic antibiotic resistance in *Lactobacilli*, and identified antibiotic resistance genes in the bacterial chromosome indicative of non-transferable and intrinsic resistance. *Lactobacilli* does not carry acquired or transmissible antibiotic resistance genes (Vizoso Pinto et al., 2006). Importantly in that context, we determined here that strain L1 does not harbor natural plasmid DNA.

The production of biogenic amines is a crucial safety criterion in the selection of probiotic strains because amines could cause health problems (Lorenzo et al., 2010). The present study has determined that strain L1 cells do not generate biogenic amines. In fact, *Lactobacillus* strains are actually considered as safe organisms particularly in terms of biogenic amine production (Arena and Manca de Nadra, 2001). Martin et al. (2005) assessed the ability of two lactobacillus strains, namely, *L. gasseri* and *L. fermentum* in producing biogenic amines and determined that none of these generate these compounds (Martin et al., 2005).

The community structure and activity of the gut microbiota co-evolve with the host from birth, and are exposed to various activities of the host genome, nutrition, as well as lifestyle. The gut microbiota regulates multiple host metabolic pathways, which results in interactive host-microbiota metabolic, signaling, and immunoinflammatory axes that physiologically link the gut, liver, muscle, as well as brain. Feeding probiotics such as *Lactobacillus* can increase the content of beneficial microorganisms (*Lactobacillus* and *Bifidobacterium*) in the intestinal tract, and inhibit the potential pathogenic microorganisms (*Salmonella* and *Escherichia coli*) to improve the intestinal microecological environment (Samuel et al., 2008; Nicholson et al., 2012). Angel et al. (2005) reported that *L. acidophilus* can improve the growth performance of broilers by improving the intestinal flora. Including *L. acidophilus* in the diet increases the *Lactobacillus* content in the ileum and cecum of broiler chicken, while the content of potential pathogenic bacteria such as *E. coli* decreases (Salarmoini and Fooladi, 2011). Further, *Lactobacillus* content increased in the feces of 1-day-old broilers after they had been fed a basic diet containing *L. plantarum* and its metabolites (Thanh et al., 2009). The present study revealed that strain L1 improves chicken growth performance and altered the composition of its intestinal microflora. Supplementation of the diet using strain L1 markedly influenced small intestinal microbial composition.

Gene products of the intestinal microflora provide enzymatic and biochemical pathways for multiple metabolic processes in the host. Turnbaugh and Gordon (2009) reported that a series of core genes encoded by the microbial genome might play a regulatory role in the host energy metabolism. For example, obese individuals carry more genes for the digestion of fats, proteins, and carbohydrates, thereby facilitating in the absorption and storage of energy from the diet compared to lean individuals (Turnbaugh and Gordon, 2009). Probiotic feeding enhances growth performance and immunity responses. The maturation of the intestinal microbiota significantly improved by probiotic feeding, yet is markedly delayed by antibiotic feeding. Probiotic feeding may thus be an intestinal health-promoting factor that may feeding efficiency during growth (Gao et al., 2017).

Gut microbiota consists of approximately 600,000 genes, i.e., 25-fold more than the number of genes in the host genome. Therefore, gut microbiota are often considered as a host organ that serves as a gut microecosystem (Lederberg, 2000; Qin et al., 2010). Such microecosystem can perform numerous metabolic functions that vary with microbiota composition. In the present study, numerous functions were determined to be involved in metabolic pathways, including the metabolism of amino acids, nucleotides, carbohydrates, energy, lipids, replication and repair, cofactors, and vitamins. All of these are probably related to the changes in the composition of gut microbiota. These findings indicate that strain L1 supplementation might promote growth performance and improve the intestinal microflora in chicken. However, additional studies are needed to confirm this.

## Conclusion

*Lactobacillus paracasei* subsp. *paracasei* L1 possesses probiotic properties such as adhesion, aggregation, hydrophobicity, as well as survival upon exposure to various gastrointestinal conditions, and lack hemolytic and decarboxylation activities. Furthermore, feeding experiments revealed that strain L1 may increase the abundance of functions related to carbohydrate and protein metabolism, and fatty acid biosynthesis in the intestinal microbiota, and improve the growth performance of chicken.

## Data Availability

The datasets generated for this study can be found in the NCBI Sequence Read Archive (http://www.ncbi.nlm.nih.gov/Traces/sra/) under the SRA Accession Number: SRP187013.

## Ethics Statement

This study was carried out in accordance with the recommendations of Guidelines for the Care and Use of Laboratory Animals, the Beijing Association for Laboratory Animal Science. The protocol was approved by the Animal Ethics Committee of the Institute of Zoology, Chinese Academy of Sciences.

## Author Contributions

LZ, YT, YX, YMT, TW, and YS performed all the experiments and wrote the manuscript. JL, HG, CW, and YC conducted the experiments and data analysis. All authors read and approved the final version of the manuscript.

## Conflict of Interest Statement

The authors declare that the research was conducted in the absence of any commercial or financial relationships that could be construed as a potential conflict of interest.

## Footnotes

1 http://ccb.jhu.edu/software/FLASH/

2 http://www.mothur.org/

3 http://qiime.org/scripts/pick_otus.html

4 http://greengenes.secondgenome.com/

5 www.i-sanger.com

## Supplementary Material

FIGURE S1 Mean proportion of the cecal intestinal microbiota and the differences in predicted functional metagenomes. Comparison of the functional pathways of microbes from the DC and DL groups at KEGG level 2 is shown.

TABLE S1 Composition of basal diets for chickens.

## Abbreviations

AGP: antibiotic as growth promoter
cfu: colony-forming units
KEGG: kyoto encyclopedia of genes and genomes
LAB: lactic acid bacteria
OTU: operational taxonomic unit
PBS: phosphate-buffered saline
PCoA: principal coordinate analysis
PCR: polymerase chain reaction.
