# Supplementary figures and images for: Probiotic Properties of Lactobacillus paracasei subsp. paracasei L1 and Its Growth Performance-Promotion in Chicken by Improving the Intestinal Microflora
Source: Front Physiol. 2019 Jul 25;10:937. doi: 10.3389/fphys.2019.00937 (PMC6670285; doi:10.3389/fphys.2019.00937)

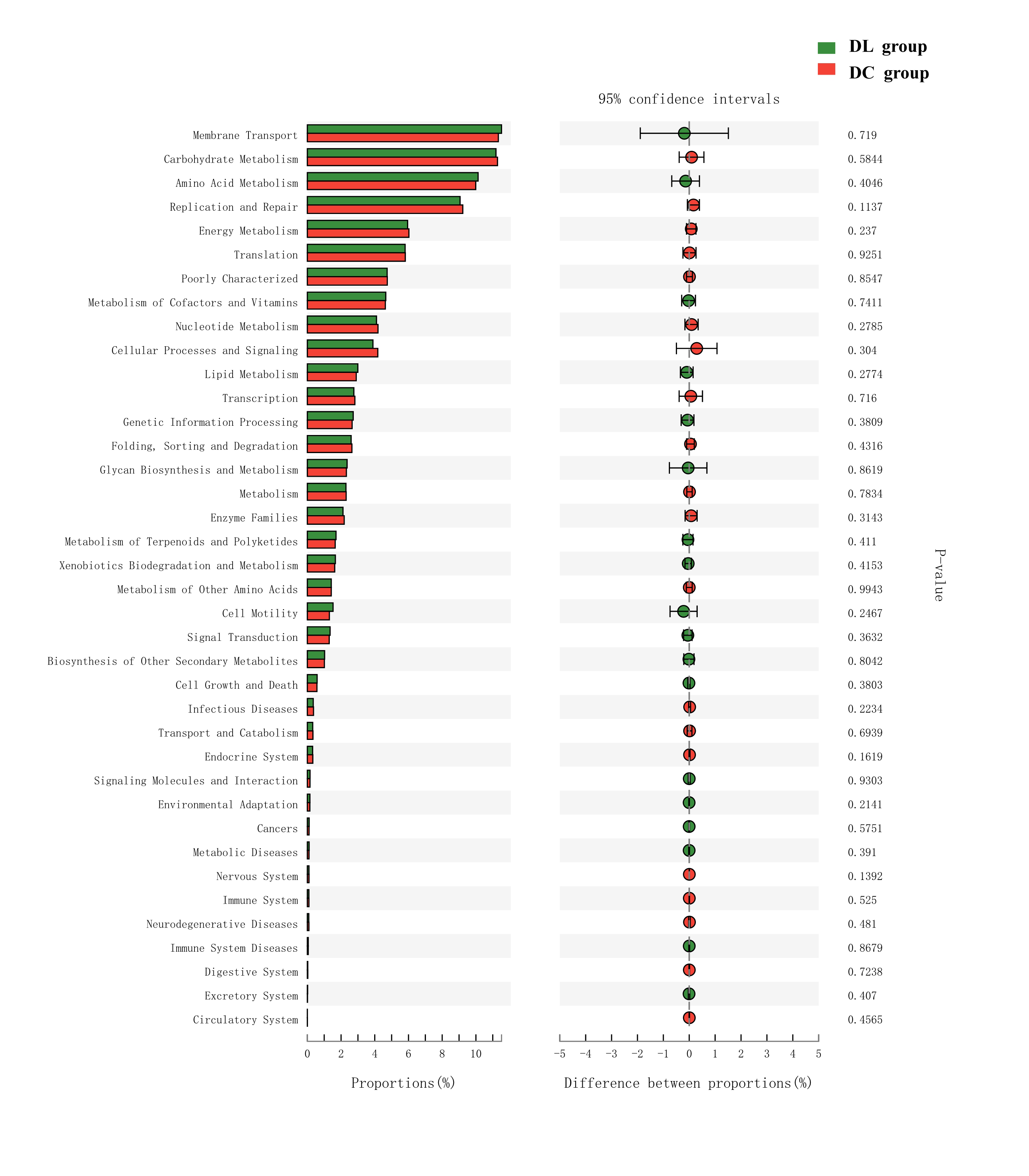

Supplement: FIGURE S1 — Mean proportion of the cecal intestinal microbiota and the differences in predicted functional metagenomes. Comparison of the functional pathways of microbes from the DC and DL groups at KEGG level 2 is shown. [file Image_1.TIF]
