# Supplementary material for: Probiotic Properties of Lactobacillus paracasei subsp. paracasei L1 and Its Growth Performance-Promotion in Chicken by Improving the Intestinal Microflora
Source: Front Physiol. 2019 Jul 25;10:937. doi: 10.3389/fphys.2019.00937 (PMC6670285; doi:10.3389/fphys.2019.00937)
Supplement: TABLE S1 — Composition of basal diets for chickens. [file Table_1.doc]

| **TABLE S1 | Composition of basal diets for chickens.** | | |
| --- | --- | --- |
| **Diet type^1^** | **8-12 W** | **13-16 W** |
| **Ingredient (g kg^-1^)** |  |  |
| Corn | 650 | 694 |
| Wheat bran | 60 | 140 |
| Soybean meal | 163 | 70 |
| Rapeseed meal | 40 | 20 |
| Cottonseed meal | 30 | 20 |
| Stone meal | 12 | 11 |
| Calcium phosphate | 12 | 12 |
| Salt | 3 | 3 |
| Trace elements and vitamin premix^2^ | 30 | 30 |
| **Calculated nutrients level** (%) |  |  |
| ME (MJ kg^-1^) | 11.70 | 11.50 |
| CP | 16.40 | 12.50 |
| Ca | 0.92 | 0.78 |
| Non-phytate P | 0.44 | 0.40 |
| Salt | 0.38 | 0.37 |
| Met | 0.503 | 0.405 |
| Lys | 1.09 | 0.94 |
| Met+Cys | 0.85 | 0.73 |
| ^1^Manufacturer: Beijing Sanyuanhefeng farming Lt. C., Beijing, China.  ^2^Detailed supplementation not disclosed by the manufacturer. | | |
